# Intercropping of marine finfish in shrimp ponds: A maiden feasibility study

**DOI:** 10.1371/journal.pone.0216648

**Published:** 2019-05-09

**Authors:** Divu Damodaran, Suresh Kumar Mojjada, Vinay Kumar Vase, Kapil Sukhdhane, Abdul Azeez P, Rajan Kumar

**Affiliations:** 1 Mariculture Division, Veraval Regional Centre of Indian Council of Agricultural Research-Central Marine Fisheries Research Institute, Veraval, Gujarat, India; 2 Fishery Resource Assessment Division, Veraval Regional Centre of Indian Council of Agricultural Research-Central Marine Fisheries Research Institute, Veraval, Gujarat, India; 3 Fishery Environment Division, Veraval Regional Centre of Indian Council of Agricultural Research-Central Marine Fisheries Research Institute, Veraval, Gujarat, India; 4 Pelagic Fisheries Division, Veraval Regional Centre of Indian Council of Agricultural Research-Central Marine Fisheries Research Institute, Veraval, Gujarat, India; 5 Crustacean Fisheries Division, Veraval Regional Centre of Indian Council of Agricultural Research-Central Marine Fisheries Research Institute, Veraval, Gujarat, India; Tanzania Fisheries Research Institute, UNITED REPUBLIC OF TANZANIA

## Abstract

Diversification of shrimp farming with marine finfish in a farmer participatory research model was attempted. The study is intended to find an economically viable finfish culture during the fallow period of shrimp farms. The Silver pompano, *Trachinotus blochi i*intercropped with Pacific white shrimp, *Litopenaeus vannamei* culture in coastal shrimp ponds was assessed for growth, survival, and economic viability. During the grow-out period of 100 days, fishes grew from 40.23 ± 1.40 g to 256.56 ± 1.08 g in weight and 12.83 ± 0.19 cm to 25.11 ± 0.09 cm in length. The daily weight gain (DWG) and daily length gain (DLG) were 2.16 g/day and 0.12 cm/day, respectively. Relative growth rate (RGR) and specific growth rate (SGR) recorded for weight was 537.80% in 100 days and 1.85% per day, respectively. Pompano has exhibited its sturdiness and adaptability to the land-based culture system as evidenced by an overall survival percentage of 89.8% including nursery and grow-out phases. The realized feed conversion ratio was 1.94. The culture period of 100 days is found to be enough to attain a desirable harvest size of 250 g. The projected production potential of the experimental demonstration farm of 4500 m^2^ water spread area for culture was 16.2 tonnes/cycle with a benefit-cost ratio (BCR) of 1.34 over operational cost. The present participatory trial empirically proved the viability of Silver pompano as an intercrop in coastal shrimp ponds. Thus, the introduction of Pompano in shrimp ponds is recommended and can be promoted for sustainable intercropping with shrimp farming along the Indian coast for improving people’s livelihoods.

## Introduction

In aquaculture, as in agriculture and forestry, the monoculture systemis at risk [1]. Aquaculture in India is dominated by shrimp farming along the country’s coastline. Shrimp farming is displaying spectacular growth in India by striking exports at an all-time high of USD 7.08 billion during the financial year 2017–18 [2]. However, sole dependence on a single species in monoculture system is not so much advisable as the country had well witnessed the catastrophe of *Penaeus monodon*. After a major setback in Shrimp farming due to viral diseases duringthe 1990s, the industry revived once *Litopenaeus vannamei* was introduced. Diversification of species and culture systems and an even distribution of production could provide resilience in the face of a changing climate and other external drivers and add economic, social and ecological insurance to aquaculture systems [1].

The projected fish production of the country by 2020 is 11.86 mmt against the recorded production of 10.8 mmt in 2016. With stagnation in the capture sector, fish farming is believed to cater for the increasing fish demand [3, 4]. Presently, Indian aquaculture is shouldered by few species of major Carps and Prawns in freshwater culture systems and *L*. *vannamei* in saline water systems. Marine finfish culture is believed to be a great investment in the saline water aquaculture sector of India [5]. But its successful and large scale adoption in the grow-out system is yet to be achieved. The possible reasons could be the degree of novelty, level of skill, technical know-how involved in open sea cage farming and most importantly lack of policy over the farming system. One of the alternatives available is to culture marine finfishes in coastal ponds, but most of the coastal ponds are currently the target of lucrative shrimp farming. Polyculture or an intercrop of marine fish during a fallow period in Shrimp farming is still an acceptable option for entrepreneurs.

The Silver pompano, a species with wider environmental tolerance, fast growth with good market demand along with a standardized seed production technology is available in India. The aquaculture of Silver pompano has been successfully established in many Asia-Pacific countries like Taiwan and Indonesia [6]. Farming of these species can be successfully carried out in ponds, tanks and in floating sea cages. The species is pelagic, very active and can acclimatize and grow well even at a low salinity of about 8‰ [5].

Intercropping is a way to increase diversity, ecological balance and effective utilization of resources and increases the quantity and quality of products along with the reduction of risk in the farming system. Crop holidays are recommended in Shrimp farming to reduce host-specific viral pathogens from culture systems which are the major health concern in Shrimp farming [7]. The practice of leaving the Shrimp farm fallow for a season is known as crop holidays and are used as a preventive measure against host-specific viral disease. Intercrop with finfish could be an alternative for crop holidays in order to reduce the build-up of host-specific viral pathogens of Shrimps without compromising the economics of the enterprise.

Although, Shrimp farming is practiced along both the coasts of India, west coast is in the developing stage with upcoming hatcheries and farm which was lacking earlier in the region. In the coastal belts of Gujarat, Surat is the hub of Shrimp farming, but it is expanding fast in other regions of Saurashtra. Majority of the upcoming farms in the region are of small to medium size (0.25 to 5 hectares) restricted to a culture period of six to seven months with a maximum of two crop harvest, leaving the farm fallow for around five to six months. Considering the duration of two months required for pond drying, plowing, and other pond management for the new season, there still exists a three to four months window for possible rearing of animals. This three to four-month window can be utilized for an intercrop of the finfish which would not only ensure the elevated farm income but also ensure sustainability. The present study was conducted to assess: (a) the economic viability of finfish (Pompano) grow-out operation during fallow period (b) the growth and survival under existing pond conditions (c) the sufficiency of short culture duration for attainment of marketable size (d) the length-weight relationship and condition factor in captivity.

The present farmer-participatory research (FPR) demonstration of Silver pompano intercropping in coastal Shrimp ponds was done at Veraval coastal belt with intent to enhance the adoption rates and payoff to research and development initiatives. The study proposes a three-year cyclic sustainable intercrop timeline design for farmers and entrepreneurs. Monoculture Shrimp farming in India and other Southeast Asian nations are frequently affected by viral disease outbreaks [8, 9], the present study was conducted to present a more stable coastal farming system to the farmers of the region.

## Materials and Methods

### Pond dimensions, nursery rearing, and stocking

The participatory culture trials were made at an existing small Shrimp farm having three grow-out ponds of 50m x 30m x 2.5 m. The farm located at Dari, Veraval, Gujarat (20°55’59.35”N and 070°19’26.63”E) is owned by Mr Bhadresh Damajibhai Bhutti. Pompano juveniles of uniform size measuring 2.8 ± 0.03 cm total length (TL)and weighing 1.6 ± 0.03 g were sourced from marine hatchery complex of Mandapam Regional Centre of Indian Council of Agricultural Research-Central Marine Fisheries Research Institute (ICAR-CMFRI). The seeds were reared in nursery hapa (1m x 1m x 1m) at a density of 1000 fishes/m^3^. Nursery hapa is a cubical meshed structure with a mesh size of 6 mm, normally used to retain fish juveniles. Two nursery hapas were installed in each Shrimp pond with ongoing Shrimp grow-out operation. After 45 days of nursery rearing, the stocking density in nursery hapa was reduced to 500 fishes/m^3^ by installing two more nursery hapas in each pond. The nursery rearing was continued for another 45 days until Shrimp harvesting. After shrimp harvest, the Pompano was released into open ponds. Fishing nets of 30 mm mesh size were used to reduce the pond dimension to 30m x 4m x 2.5m. The effective stocking density of 10 fishes/m^3^ was maintained for the on-farm demonstration trials, andthe water level of 1.5 m was maintained throughout the culture operation. Groundwater bores were used as a source of water with salinity ranging between 15 and17 ppt during the culture period. The fishes were handled ethically during packing, transportation, stocking, and rearing.

### Feed and feeding management

Commercially formulated extruded floating pellet feed (Growel Feeds Ltd, Andhra Pradesh, India) was used for the entire culture to avoid feed wastage and ensure fair farm economics, better water quality, andavoid extra deposition of organic waste in thepond bottom. Circular, floating feeding rings made of high-density polyethylene (HDPE) were placed and feed was broadcasted in the rings to avoid uneven feed disbursement and wastage throughout the pond. Feed with different pellet size and proximate composition were used during the grow-out period at different feeding rates and rations (Table 1). Fishes were fed at 08:00 and 17:00 IST daily. The feed ratio was adjusted based on biomass estimated on each sampling days.

**Table 1:** Feed, proximate composition, and feeding rates of *T*. *blochii* during farming experiments.

Pellet feed (size in mm)	Fish size (g)	Protein (%)	Fat (%)	Fibre (%)	Moisture (%)	Feeding rate (% of biomass/day)	No. of feedings/day
Starter II (1.8 mm)	<100	45	10	2.5	11	4.0	2
Grower I (3.0 mm)	100–150	40	10	3.5	11	3.5	2
Grower I (3.0 mm)	> 150	40	10	3.5	11	3.0	2

Other feed ingredients include: crude fibre: 2.5–5.0% (max.); crude ash: 15.0% (max); calcium: 2.0% (min.); phosphorus: 1.5% (min.); moisture: 5.0–8.0% (max.); mineral and vitamin premix

### Grow-out and farm management

Minimum maintenance of one-hour daily aeration was done using two-paddle wheel aerators (1.5hp) at 04:00 IST and 10% water exchange per month during grow-out operations. Pellet feed of suitable size was selected based on the mouth size of the fish. Pellet sizes were kept below the vertical mouth size (MS) which is estimated as MS = (UJ^2^ + LJ^2^–2*UJ*LJ*Cos 30^0^)^1/2^ at gape of 30^0^ where LJ and UJ are the lengths of the upper and lower jaw, respectively [10, 11]. Water quality parameters were monitored every 10^th^ day during the culture period. Key water quality parameters like temperature, pH, salinity, dissolved oxygen, turbidity, and ammonia were monitored and analyzed using a handheld multi-parameter kit (YSI professional plus, USA) and standard protocols [12]. The water samples were collected just before the sunrise on sampling days.

### Estimation of growth parameters

Sampling was done at intervals of 10 days over a grow-out operation of 100 days using cast net (30mm mesh size) to assess the well-being and growth of the fish. Mean total length (nearest mm) and mean weight (nearest 0.1 g) were estimated for each sampled fish. Length-weight relationship of a fish is usually expressed by the equation, W = aL^b^[13] where W is body weight (g), *L* is total length (cm), *a* is the coefficient related to body form, and *b* is an exponent indicating isometric growth when equal to 3 [14]. The parameters *a* and *b* were estimated by the least-square method from logarithmically transformed length and weight attributes and the association degree between weight-length variables was assessed using a correlation coefficient (*R*^2^).

Fulton’s condition factor (K = W*100/ L3; where W and L are observed weight and length) was estimated to assess the condition of individual sampled fish [15]. Observed weight is also compared against the estimated weight from the length-weight relationship established from wild-caught specimens in order to assess the growth performance in captivity against their wild counterpart [16].

Growth performance parameters *viz*., daily weight gain (DWG), daily length gain (DLG) relative growth rate (RGR), specific growth rate (SGR), and feed conversion ratio (FCR) were also estimated as given below to evaluate the quality of growth [17].

DWG=(W2−W1)÷(t2−t1)

DLG=(L2−L1)÷(t2−t1)

RGR(weight)=(W2−W1)×100÷W1

RGR(length)=(L2−L1)×100÷L1

SGR(weight)=[lnW2−lnW1]×100÷(t2−t1)

SGR(length)=[lnL2−lnL1]×100÷(t2−t1)

FCR=totalfeedintake÷totalweightgain

W2 and W1 are final and initial weight;L2 and L1 are the final and initial length and t2 –t1is the time lag.

The harvest is reported as an average of three replicates. The profit was estimated as the difference between total revenue generated and total operational cost. The benefit-cost ratio (BCR) was estimated as the ratio of total revenue generated and the total operational cost incurred. The production potential was estimated by extrapolating the production achieved per unit volume in the present trial to the total grow-out volume available at farm.

### Timeline design for trial

**Fig 1:**
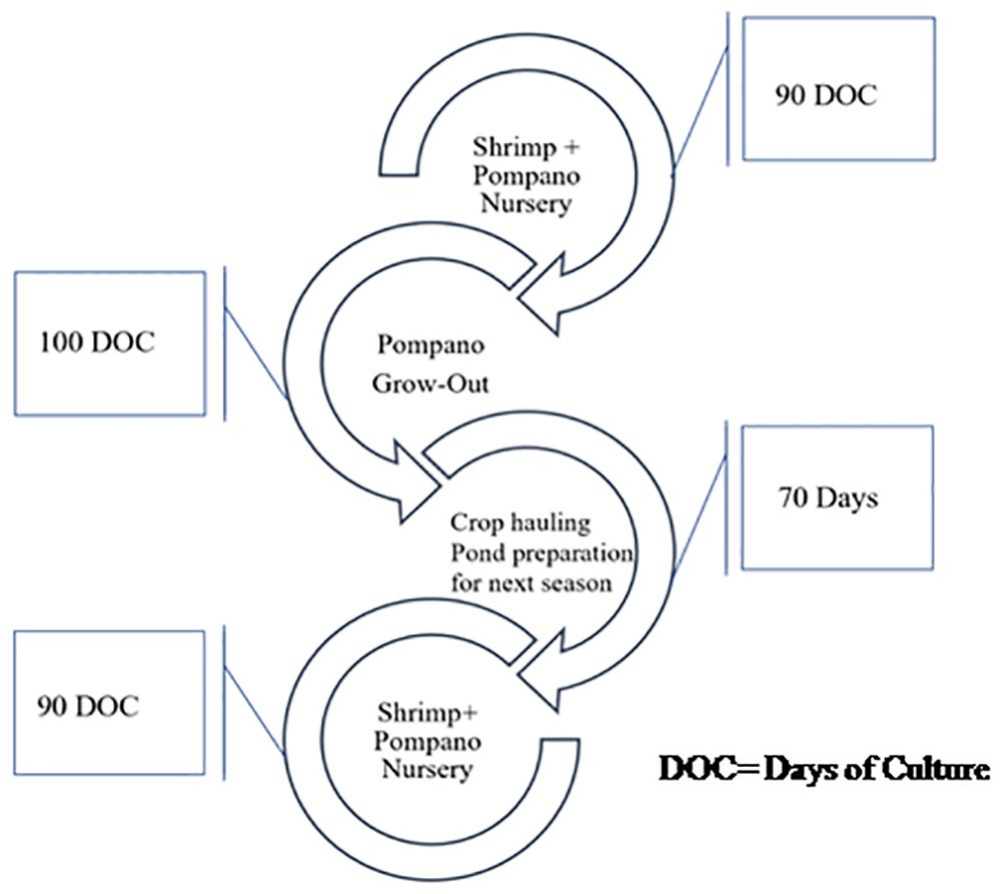
Schematic pompano intercropping in shrimp farms opted for trial.

Timelines are significant in assessing the feasibility of any project. In order to work out the most viable and sustainable intercropping seasons, species and culture window, a timeline was designed, considering the overall amount of time fixed. The alternating cropping with fair culture duration and crop overhauling periods were organized in timelines (Fig 1). The concept of intercropping with sustainability was considered as a critical concern. Altering the crops with enhanced farm economics was also taken into account while finalizing the timeline strategy.

### Statistical analysis

The thirty fishes from each pond were recorded on sampling days. The extreme outliers were removed after initial screening using box plots. The variance showed no significant differences across replicates, thus parametric test was performed on the data. The mean lengths and weights were compared across replicates using a One-Way ANOVA. As no significant differences (p < 0.05) across replicates were observed, a pooled mean for length and weight along with standard error (mean ± SE) were reported graphically across days of culture. The DWG, DLG, SGR, and RGR were estimated based on average lengths and weights (pooled for replicates). The slope of the length-weight relationship was tested against isometry (b = 3) using the Student’s t-test. The environmental parameters estimated from three ponds (replicates) were presented as mean ± SD. The coefficient of variation (CV) was also estimated to visualize the extent of fluctuation during the entire culture duration. The tables, graphs, and data analysis were done using MS-Excel and Statistica ver. 8.0 of Statsoft Inc, USA [18].

### Ethical statement

The study was approved and periodically assessed by the Institute Research Council (IRC) of ICAR-Central Marine Fisheries Research Institute. The manuscript was approved for submission considering set criteria including ethical aspects by Priority Setting, Monitoring and Evaluation (PME) cell of ICAR-Central Marine Fisheries Research Institute.

## Results

### Growth, production and production potential

**Fig 2:**
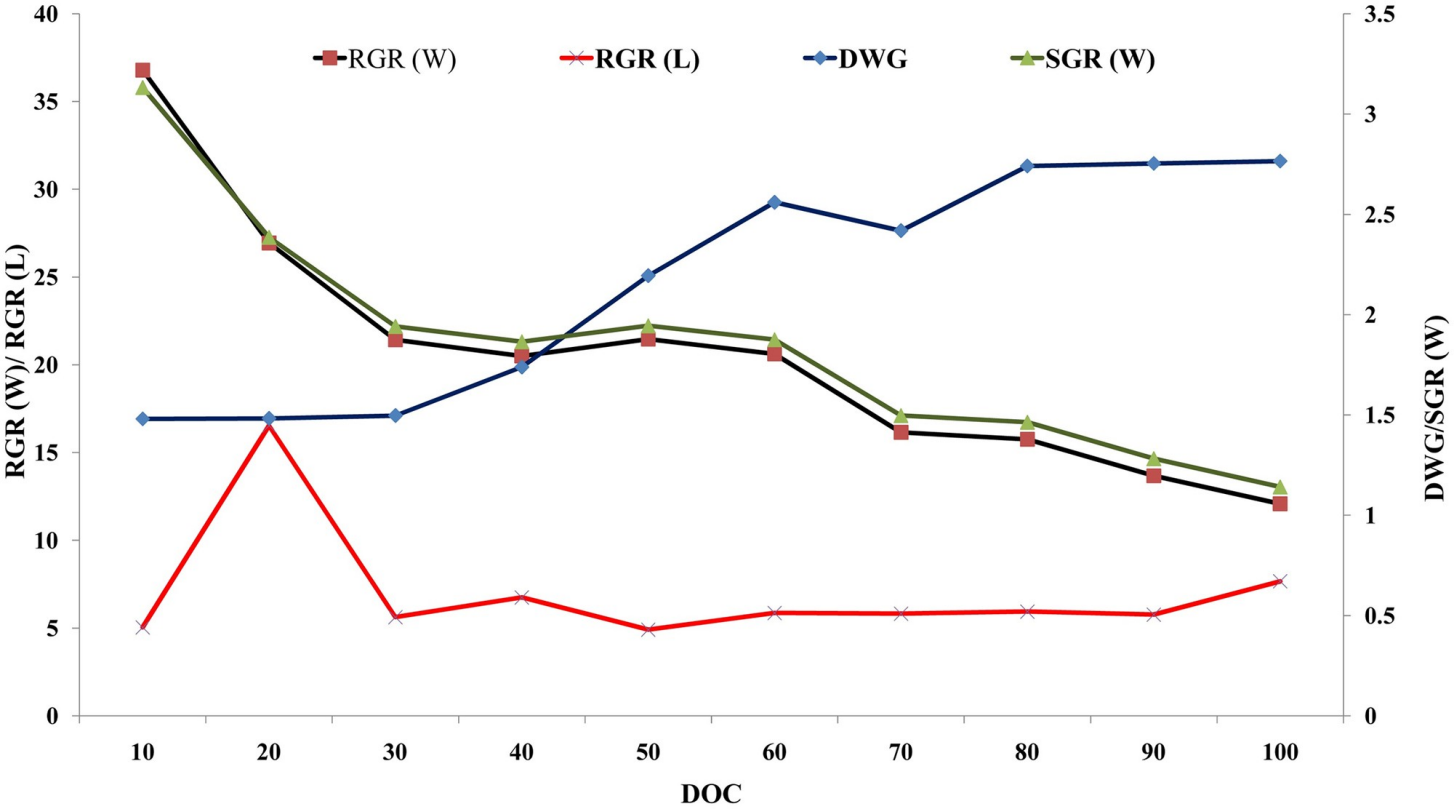
Temporal variation of growth indices across the grow-out culture operation.

The fishes grew from 40.23 ± 1.40g to 256.56 ± 1.08 g in weight and 12.83 ± 0.19 cm to 25.11 ± 0.09 cm in length during the grow-out period of 100 days. The average length and weight across replicates on all sampling showed no significant difference (p<0.05). The daily weight gain (DWG) and daily Length gain (DLG) were 2.16 g/day and 0.12 cm/day, respectively during the period. Relative growth rate (RGR) and specific growth rate (SGR) for weight was 537.80% in 100 days and 1.85% per day, respectively. Similarly, RGR and SGR for length were 95.69% and 0.67% per day, respectively, for the 100-days of culture. Nevertheless, the above-estimated parameters were not constant thought-out the culture duration. DWG showed an increasing trend as the culture progressed whereas the reverse trend was observed for RGR and SGR for the weight (Fig 2). The RGR (L) showed an increasing trend during the initial period of 20-days of culture (DOC), later sharply declined until 30 DOC. Subsequently, it exhibited a steady increment during the rest of the culture period. On the contrary, the DWG showed an increasing trend, which reached a maximum value on 60 DOC and later declined for a short period (10 days). The daily growth rate became stable after 80-days of culture.

**Fig 3:**
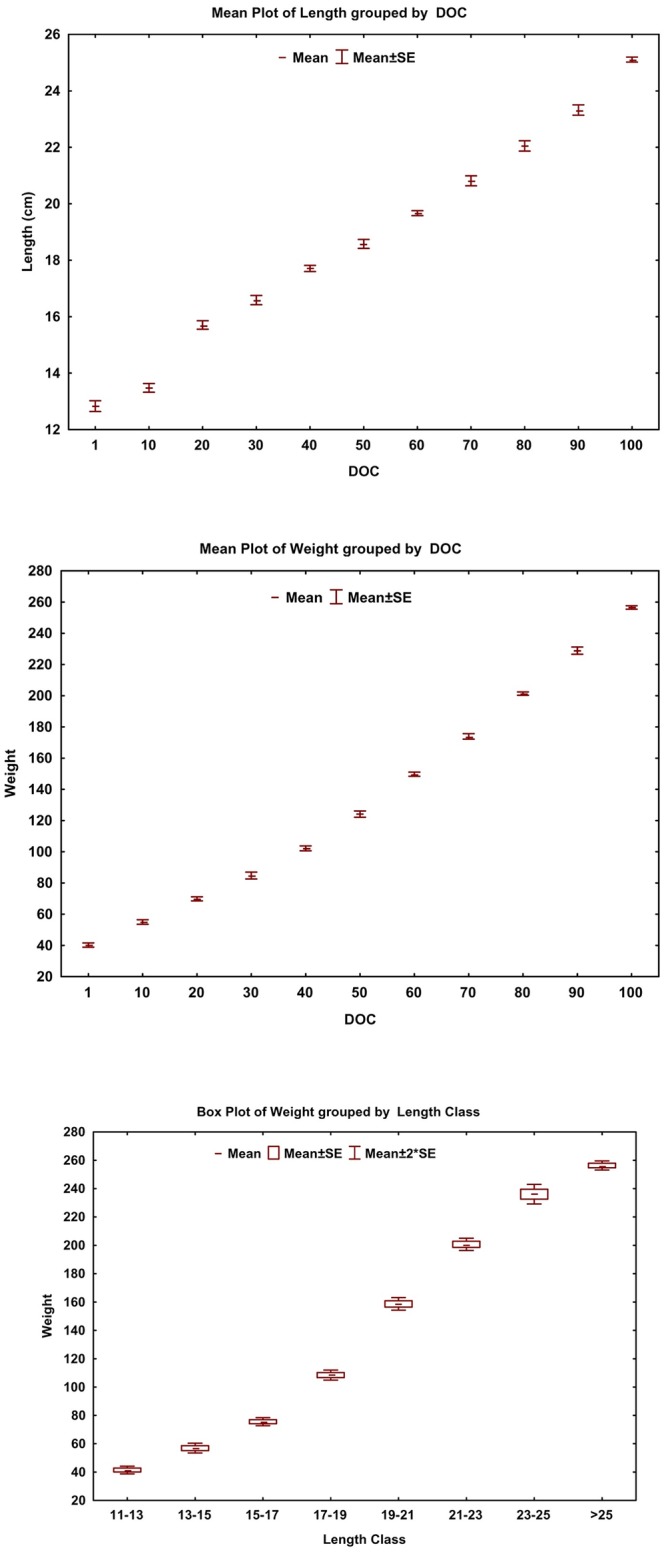
Length and weight progession of Silver pompano and mean weight across different length class during 100-days of grow-out culture.

The increase in length in the initial 10-days of grow-out operation was minimal as reflected in very smooth slope during the period whereas rapid weight gain was observed during the same period. The rate of weight gain was relatively high post 50-days of grow-out, reflected in a steeper slope of growth in weight when plotted against DOC (Fig 3a and 3b). The estimated FCR for the grow-out operation was 1.94 which is quite reasonable, for economic farming practice.

The plot of weight against length class showed a wider dispersion among length class (17–25 cm) whereas the weight of individuals after attaining > 25 cm showed the least variation leading to more uniform sized final harvest (Fig 3c), which was a desirable output for the enterprise. An average harvest of 460 kg and revenue of USD 1973 from 120 m^2^ water spread area (each replicate) was achieved during the trial. The average operational cost including feed and seed was around USD 1476/ replicate giving anaverage profit of USD 496.8. Production potential of the farm (3grow-out ponds) was estimated at 16.2 tonnes/cycle (7.2kg/cycle/m^3^) with a BCR (over operational cost) of 1.34. The operational costs other than feed and seed were considered at par with Shrimp farm as mentioned by the entrepreneur to calculate the BCR. An additional income for the farm from 100-days Pompano intercrop was projected to be around USD 14,583 (Table 2).

**Table 2:** Operational cost and economics of the trial and projected farm economics.

Particulars	Experimental Trial	Projected for Shrimp farm
Pond dimensions (mxmxm)	30x4x1.5	(50x30x1.5) x 3
Stocking density (Nos/m3)	10	10
Stocking No.	1872	67500 (22500x3)
Grow-out size (kg)	0.256	0.25
Survival (%)	96%	96%
Harvest (kg)	460	16200 (5400x3)
FCR	1.94	1.94
Selling price (USD/kg)	4.29	3.57
Feed cost (USD)	961.47	33883.89
Seed cost (USD)	85.6 (2000 x 0.0428 USD)	2996 (70000 x 0.0428USD)
Other operational cost* (USD)	428.91	6433.65
Revenue (USD)	1972.99	57902.85
Profit (USD)	496.82	14582.94
BCR (over operational cost)	1.34	1.34

1 USD = Rs. 69.94 INR

*Based on the entrepreneur’s monthly operational cost of Shrimp farm

### Length-weight analysis and condition factor

**Fig 4:**
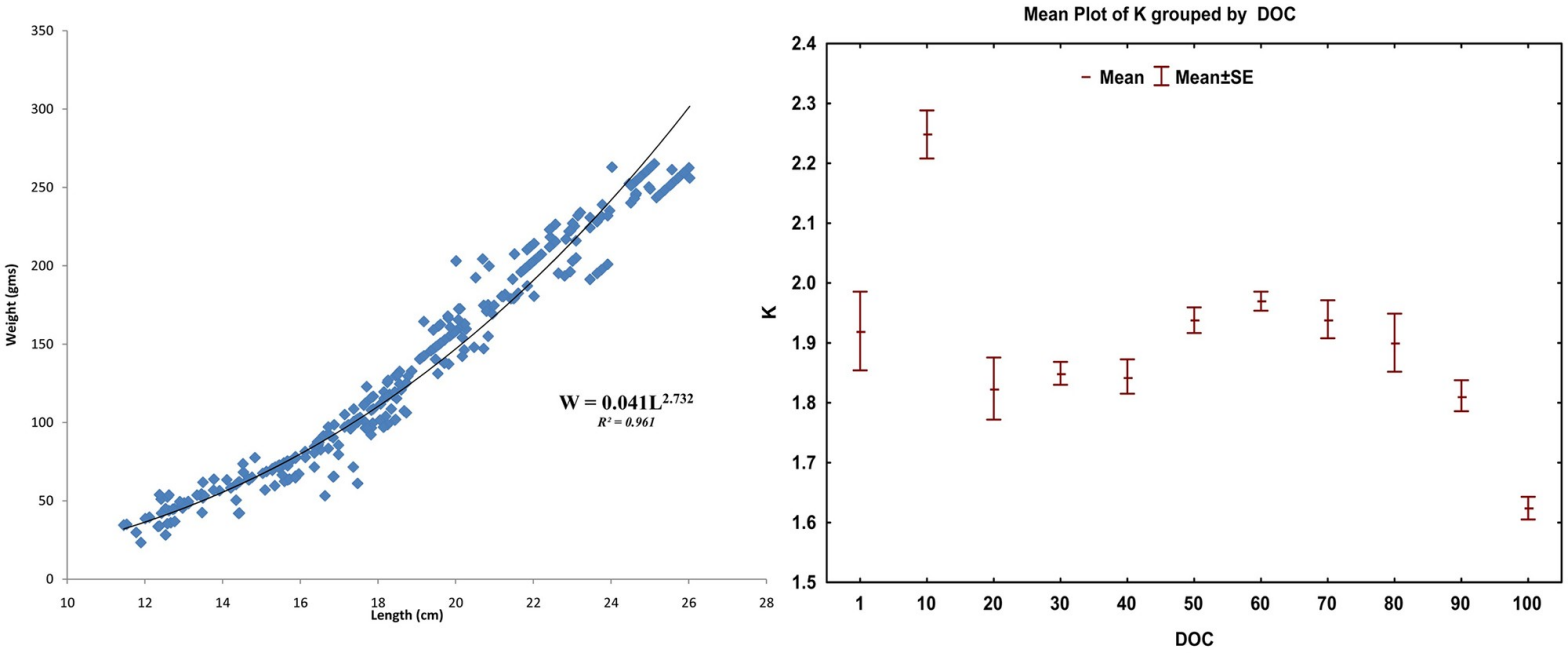
The length-weight relationship and variation in condition factor (K) during growout operation of Silver pompano.

The length-weight relationship (LWR) for the species under culture conditions was estimated as W = 0.041*L^2.732^(R^2^ = 0.961) (Fig 4a). The growth was found to be hypoallometric when tested against isometry (Student’s t-test, t = 8.82, df = 328, p < 0.01). The LWR for a species reflected the progression of weight gain as the individual grew in length. The condition factor (K), an index of the quality of growth of the species, ranged from 1.14 to 2.84, with 94.8% cases having a value greater than 1.5. Nevertheless, there exists a pattern in condition factor across the culture timeline. The condition factor showed a decreasing trend post 60 DOCwhere the lowest K was recorded on harvest day (Fig 4b).

### Water quality parameters and survival

The temperature, salinity, turbidity, DO, pH and NH_3_-N recorded on sampling days across replicates were 26.6 to 32.2°C, 15.0 to 17.2 ppt, 35 to 43 NTU, 4.8 to 5.5 ppm, 7.8 to 8.8, and 0.024 to 0.051 ppm, respectively. Ammonia was the most variable parameter (CV = 26.3%) whereas temperature (CV = 2.97%) was least variable across the sampling days (Table 3). None of the recorded parameters were beyond the tolerance limit of the animal on any sampling days ascertaining the reasonable culture condition provided during grow-out operation.

**Table 3:** Key water quality parameters recorded during the culture period.

DOC	Temperature (°C)	Salinity (ppt)	Turbidity (NTU)	DO (ppm)	pH	Ammonia (10^−2^ ppm)
1	29.6 ± 0.20	15.0 ± 0.26	41 ± 0.62	5.5 ± 0.22	7.8 ± 0.29	4.5 ± 0.23
10	30.1 ± 0.61	16.1 ± 0.35	42 ± 0.70	5.4 ± 0.15	8.1 ± 0.26	3.2 ± 0.15
20	30.4 ± 0.36	15.0 ± 0.31	39 ± 0.78	5.2 ± 0.20	7.9 ± 0.21	4.2 ± 0.41
30	31.2 ± 0.56	15.4 ± 0.38	35 ± 0.82	5.3 ± 0.19	8.6 ± 0.28	2.9 ± 0.28
40	31.8 ± 0.48	16.1 ± 0.29	40 ± 0.61	4.8 ± 0.23	8.2 ± 0.35	2.7 ± 0.44
50	32.0 ± 0.33	17.2 ± 0.54	37 ± 0.69	5.1 ± 0.18	8.4 ± 0.30	5.0 ± 0.21
60	30.4 ± 0.53	15.5 ± 0.28	43 ± 0.53	5.2 ± 0.23	8.5 ± 0.39	3.5 ± 0.16
70	31.6 ± 0.66	15.6 ± 0.37	42 ± 0.79	5.4 ± 0.17	8.2 ± 0.29	2.4 ± 0.26
80	32.1 ± 0.98	16.8 ± 0.32	40 ± 0.96	5.3 ± 0.23	8.3 ± 0.33	4.8 ± 0.22
90	32.0 ± 0.39	17.0 ± 0.23	37 ± 0.87	5.5 ± 0.38	8.8 ± 0.31	5.1 ± 0.21
100	32.2 ± 0.57	16.5 ± 0.3	38 ± 0.56	5.4 ± 0.26	8.3 ± 0.27	3.8 ± 0.14

The mean percentage survival during the ninety-day retention of seed in nursery hapa with ongoing Shrimp farming with feeding and cleaning as only management practices was as high as 93.6%. Overall survival of 89.8% was recorded over an entire period of 190 days (nursery and grow-out).

## Discussion

The maintenance of water quality parameter in a culture system is imperative for successful farming practices, as it has a strong bearing on the survival and growth performance of the cultured organisms. Although Silver pompano is one of the sturdiest among candidate cultivable finfish species which are commercially used for farming, a suitable culture environment must be provided for good growth and survival. Most of the recorded water quality parameters on sampling days were well within the tolerance limit of the species and were similarto other reported land-based trials [5, 6]. The temperature was the least variable environmental parameter attributed to the high specific heat of water, large sized pond with adequate water depth and short culture duration (minimum climatic change). The NH_3_-N was the most variable water quality parameter possibly due to minimum aeration provided during the culture period. Nevertheless, the concentration of NH_3_-N never became sub-optimal during the culture period and hence had no indicative impact on the growth and survival of the cultured species. The recorded percentage survival of Silver pompano during the entire retention period of 190 days (nursery and grow-out) was 89.8%, which was in line with the pond culture trial results (91.32%) reported at Andhra Pradesh [6]. The good survival emphasized the sufficiency of pond management practices and proved the sturdiness of Pompano and its ability to adapt to the land-based culture system. Higher survival rates between 95–100% have been recorded for the species but from open sea cages [19–21]. The survival of other cultured Carangids like *Trachinotus ovatus* [22], *Mugil cephalus*, *Chanos chanos* etc. in brackish waters were not at par with Silver pompano making it the most favored species for culture in diverse culture systems and locations.

DWG and SGR reported in the current study are comparable with earlier studies [6]. The growth in weight for similar length-class [6] in their study was estimated at 2.07g/day (DWG) and 1.42% SGR, which was lower than the growth observed during our trial. Though the performance of the species in ponds during the current study is similar or better than previous trials in coastal ponds [6], their growth performances were much lower than that recorded from cages. In open sea cages, Silver pompano recorded SGR and DWG of more than 2.5 [19–21] which further offers the scope for growth optimization in the land-based closed farming system. This study reports an FCR value of 1:1.94, which is slightly higher than 1:1.83 reported by [6]. A possible explanation could be the absence of dedicated fertilization of the pond for augmenting natural productivity unlike in the earlier case where such pond preparation schedule was followed.

The minimal increment of length for the first 10-days of grow-out with rapid weight gain over the same period could be due to space constraintduring their retention in nursery hapa. The constraint of space was alleviated once the fishes were released in an open pond with more space and elevated feeding rate. Similar inference can be made from the length progression graph as the prominent rise in the condition factor against 10^th^ DOC. Similar reduced increment in length was not evident in the growth progression table presented by [6], as they used a lower stocking density and higher feeding rate during nursery rearing in hapa. A higher stocking density with sub-optimal feeding in present study induced stunting which could be utilized to achieve marketable-sized fish in a smaller time frame. A definite declining trend in condition factor post 50 DOCcan be explained bythe ascending slope of RGR (L) and descending slope of RGR (W).

The mean weight against DOC reflected the growth of species, which was not uniform for all the fishes stocked in the ponds. Nevertheless, the reduction in dispersion in weight during the terminal phase of culture was a positive culture attribute, leading to uniform size harvest of 250 g size fishes. This is a highly preferable size group by clients/buyers especially for restaurants where it acts as a substitute for Silver pomfrets for which the given size of 250 g is the very common serving.

The estimated length-weight relationship, W = 0.041*L^2.732^, indicated a hypoallometric progression of weight as individual grew in length, which was within the range (2.25 to 2.85) of earlier studies from the wild [16, 23]. But this alone does not give an indication of the quality of growth the organism experienced. The most widely used well-being index for fishes is the condition factor, which is derived from the length-weight relationship. The ratio of the observed weight against the weight of the same sized (length) isometrically grown fish is known as the condition factor for that fish. The condition factor of 1.0 or above indicates good growth [24–26]. Majority of the estimated K values (94.8%) in the present study were above the value of 1.5 indicating the favorable culture conditions provided to the fishes to achieve good growth. The growth in a natural system (LWR), like in open seas, can be used to estimate the standard weight which can be compared with weight gain in captivity to evaluate the growth realization in rearing systems. The LWR reported in [16] was used to estimate the standard weight for the equivalent length of the sampledfish. The proportion of recorded sample above the standard weight estimated from LWR from the wild of similar climatic regime could be used as an index of growth in captivity. In the present study, almost 97% of the recorded weights were above the estimated standard weight reflecting the good growth recorded in present captive rearing conditions. An evident decline in the slope of the DWG curve post 80 DOC and weight curve against terminal length classes were indicative of the onset of slow growth asking for economic harvest. The stocking density of 10 fish/m^3^ for 100 days exploited the exponential growth phase (in weight) and was suitable where smaller culture window of 3 to 3.5 months was only available.

As the culture trial was made in collaboration with a young and progressive Shrimp farmer, an attempt was made to evaluate the economic performance of the 3 months Silver pompano intercrop. In the current experiment, an economically viable BCR (over operational cost) of 1.34 was realized. The farm consists of three ponds of similar dimensions and hence a farming projection was also expedited. The feed and seed costs were extrapolated from the present experimental trial. The other operational costs including watch and ward, and electricity were taken at par with Shrimp farming based on the entrepreneur’s current farming expense records. A total projected profit generated by adopting this model could be around USD 14,583in 100 days which appears quite lucrative even ata nominal selling price of USD 3.57/ kg. The harvest coincided with the fishing ban in the region which offered ample opportunity for even a higher price realization than used for estimation of present BCR. Also, such initiatives reduced the risks associated with single species farming and added income almost similar to Shrimp in a sustainable manner from existing resources.

Silver pompano was observed to exhibit an omnivorous character, scavenging the Shrimp farming ponds waste and productivity as their natural feed which is a desirable attribute for co-culture. Nevertheless, co-culture of Shrimp and Pompano was avoided as crustaceans form the natural diets of Pompano and could pose threat to the standing Shrimp stock in the culture pond.

### Timeline for sustainable intercrop

**Fig 5:**
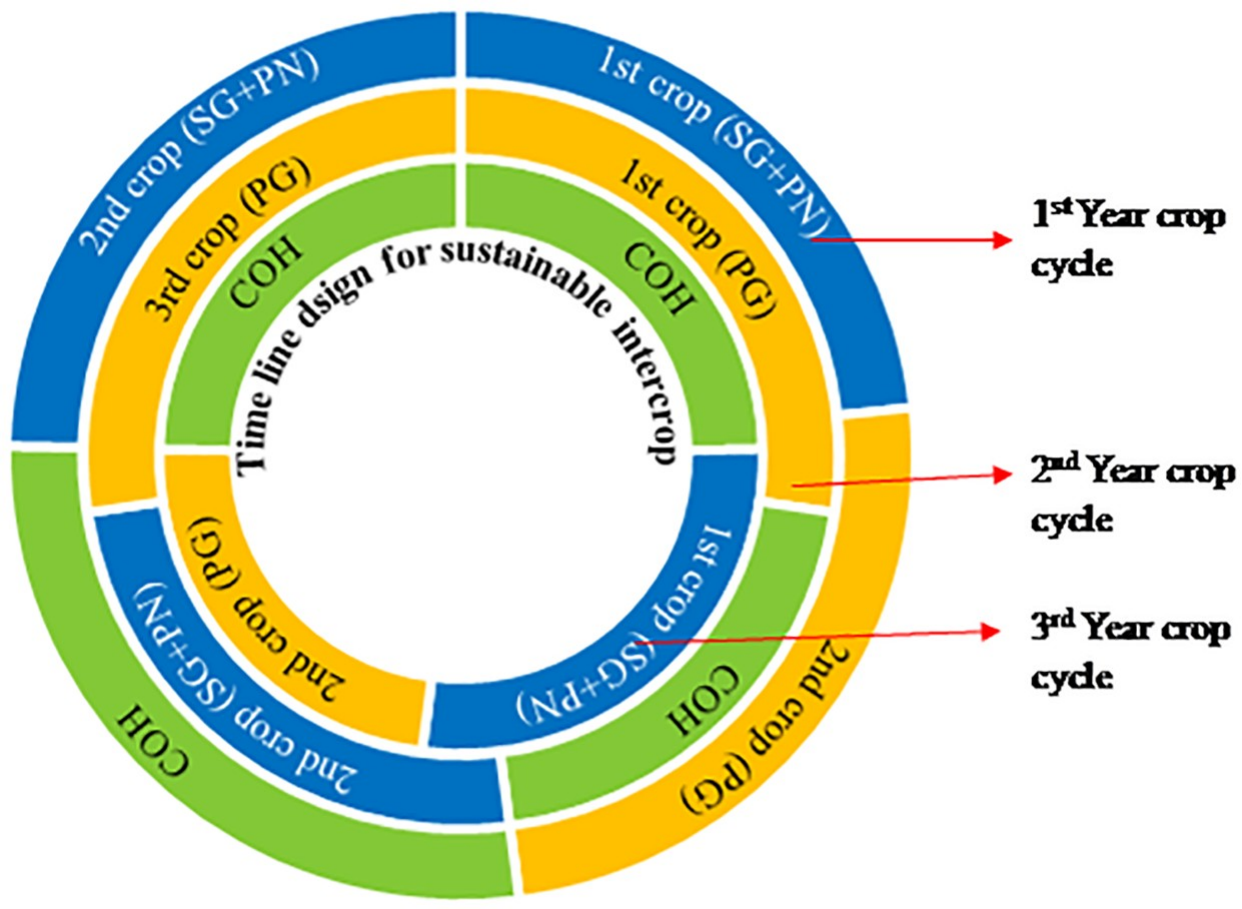
Proposed 3-year cyclic and sustainable intercropping farming system of Silver pompano in coastal Shrimp farms. Crop Over Hauling (COH); Shrimp Growout +Pompano Nursery (SG+PN); Pompano Nursery (PN).

The current study was intended to explore the economic viability of Silver pompano as an intercrop in small and medium Shrimp farms. The economics were found to be viable and at par with Shrimp farming for the small/medium farms. Intercropping in the farming system increases sustainability and stabilizes farm economics by reducing vulnerability to factors like climate change, market instability, and disease outbreak. The intercrop of finfish similar to crop holidays (absence of Shrimps from farm) helps in reducing the build-up of host-specific Shrimp virus from the system rendering the following Shrimp crop less vulnerable to viral diseases. The scavenging of organic waste by finfishes like Pompano also ensures lower bacterial load in a culture system and hence reduces bacterial disease outbreaks [27]. Through the results obtained from the experimental demonstration and interactions with local entrepreneurs, a strategic timeline is being proposed for the sustainable intercropping of Silver pompano in Shrimp farms in order to make coastal farming a sustainable and farmer-friendly. The three-year cyclic farming model (Fig 5) consists of eight grow-out operations (4 Shrimp and 4 Pompano in alteration) instead of the earlier six grow-out operations of Shrimps. Although the proposed model reduces total Shrimp production from the farm, the overall production of the farm will be elevated. In addition, the maintenance and risks during Pompano culture will be substantially low as it is among the sturdiest fishes. In absence of crop insurance mechanism in the Indian aquaculture industry, such intercropping models may be the ideal farming practices especially when the scale of operations is small or medium.

## Conclusions

The Shrimp farming practice along the northwest of India is in the developing phase and mostly operates at small to medium-scale with a minimum of 3–4 months of fallow period. This unutilized period offers an opportunity to introduce a sturdy marine finfish as an intercrop in these Shrimp ponds. The Silver pompano with higher survival, fast growth rate, and smaller grow-out period is found to be a suitable candidate. The economic viability of Pompano intercrop favors its adoption by the Shrimp farmers of the region. The intercrop will ensure year-round operation of the farmand additional income by utilizing the present fallow period. The study could be a model for adoption in other southeast Asian countries where crop holidays are practices leaving the farm idle for a part of the year.
